# Varicella Zoster Virus–Associated Meningoradiculitis in an Elderly Patient: A Case Report

**DOI:** 10.1155/crdi/9461187

**Published:** 2026-10-04

**Authors:** Yi Ru Tan, Samuel Zheng Fong Goh, Yeow Hoay Koh, Gabriel Shih Chung Yiin

**Affiliations:** ^1^ Department of Neurology Changi General Hospital Singapore cgh.com.sg

**Keywords:** case report, immunocompetent host, meningoradiculitis, varicella zoster virus, zoster paresis

## Abstract

**Introduction:**

Motor complication in immunocompetent individuals with varicella zoster virus (VZV) reactivation is uncommon and may mimic a compressive spinal disorder.

**Case Presentation:**

A 77‐year‐old immunocompetent woman presented with an acute left foot drop preceded by coryzal symptoms and lower back pain. A vesicular rash developed over the left foot one week after the weakness began. Examination showed a predominantly severe left ankle dorsiflexion weakness with reduced sensation over the L5 and S1 dermatomes. A noncontrast magnetic resonance imaging (MRI) of the lumbosacral spine showed noncompressive degenerative changes. Cerebrospinal fluid (CSF) analysis showed lymphocytic pleocytosis, elevated protein, a reduced CSF‐to‐serum glucose ratio, and isolated VZV DNA on the polymerase chain reaction (PCR). VZV DNA was also detected from a vesicle swab. She received 14 days of intravenous acyclovir with neuropathic analgesia. A nerve conduction study and electromyography (NCS/EMG) at three months showed a severe left common peroneal neuropathy with sciatic nerve involvement. There was an initial slow motor recovery with persisting neuropathic pain, but only a residual moderate ankle dorsiflexion weakness remained at 9 months.

**Discussion:**

The combination of preceding radicular pain, vesicular rash, significant leg weakness, and positive VZV DNA in CSF and vesicle specimen supported a diagnosis of VZV‐associated meningoradiculitis. The NCS/EMG was consistent with an evolution of VZV involvement at multiple neuraxial levels into a predominantly peripheral neuropathy. This diagnosis is easily missed when radicular pain precedes or occurs without a rash and when imaging is disproportionate to the severity of deficit. CSF PCR and skin lesion sampling are central to an early diagnosis.

**Conclusion:**

VZV should be considered in patients with acute painful radiculopathy with significant limb weakness that is out of proportion to imaging findings, even without immunosuppression or an early rash. Early antiviral therapy can limit disability, although residual neurological deficits and pain may persist.

## 1. Introduction

Varicella zoster virus (VZV) reactivation typically presents as a painful dermatomal rash (herpes zoster), but peripheral nervous system involvement such as sciatic or common peroneal neuropathy is uncommon, especially in immunocompetent individuals. We report an elderly woman with VZV‐associated meningoradiculitis to emphasize the diagnostic challenges and importance of early antiviral therapy.

## 2. Case Report

A 77‐year‐old woman with a background history of hypertension, knee osteoarthritis, and osteoporosis presented to the emergency department (ED) with acute left foot weakness. Three weeks before admission, she developed low‐grade fever, headache, and coryzal symptoms shortly after receiving an influenza vaccination. These symptoms resolved, but she subsequently experienced persistent left lower back pain and leg cramps. One week prior to admission, she was assessed and initially diagnosed with lower back strain in the ED visit and was discharged with oral analgesics. Weakness of left ankle dorsiflexion began at about this time, approximately one week before the rash appeared. A day before the current admission, she developed numbness and itchy rashes with small blisters over the dorsum of her left foot. There was no history of preceding trauma, constitutional symptoms, or incontinence. She recalled a history of chickenpox infection during adolescence but was not on any immunosuppressant. A timeline of the clinical course is provided in Table 1.

**TABLE 1:** Timeline of the patient’s clinical course.

Time point	Event
Three weeks before admission	Low‐grade fever, headache and coryzal symptoms shortly after influenza vaccination; these symptoms resolved
Subsequent weeks	Persistent left lower back pain and leg cramps
One week before admission	Onset of left ankle dorsiflexion weakness. Assessed in the emergency department, diagnosed as lumbar strain and discharged with oral analgesia
One day before admission	Numbness and an itchy vesicular rash over the dorsum of the left foot (approximately one week after the onset of weakness)
Admission (Day 0)	Severe left foot drop (ankle dorsiflexion MRC Grade 0) with L5‐S1 sensory loss, moderate weakness of ankle plantar flexion/inversion and eversion (MRC Grade 3), and mild weakness affecting the left hip flexion/knee flexion and extension (MRC Grade 4). CSF showed lymphocytic pleocytosis with varicella zoster virus (VZV) DNA positive on PCR; VZV DNA was also detected from a vesicle swab. Diagnosis of VZV meningoradiculitis was made
Days 0–14	Intravenous acyclovir for two weeks with neuropathic analgesia, and then transferred to a rehabilitation ward for physiotherapy and gait training
Three months (follow‐up)	Nerve conduction study and electromyography (NCS/EMG) showed a severe left common peroneal neuropathy at the fibular head with sciatic nerve involvement, without electrophysiological evidence of L5 radiculopathy. Needle EMG of the left tibialis anterior revealed acute spontaneous responses with polyphasic discrete motor unit action potentials, suggesting residual axon continuity as well as ongoing renervation
Five months (follow‐up)	Partial motor recovery with improved gait; neuropathic pain persisted.
Nine months (follow‐up)	Lower limb strength returned to full power in all muscle groups except ankle dorsiflexion, which remained at MRC Grade 3.

On examination, she was afebrile and hemodynamically stable. There were clusters of vesicular rashes over the left lateral shin, dorsum of foot, and left big toe, consistent with a dermatomal distribution (Figure 1). The cranial nerves and upper limbs were normal. The tone was normal in the lower limbs. There was a severe weakness in the left ankle dorsiflexion with Medical Research Council (MRC) Grade 0, moderate weakness of ankle plantar flexion/inversion and eversion (MRC Grade 3), and mild weakness affecting the left hip flexion/knee flexion and extension (MRC Grade 4). There was a reduced left ankle jerk but normal knee jerks. Plantar responses were flexor bilaterally. There was reduced pinprick sensation over L5 and S1 dermatomes. There was no cerebellar sign.

**FIGURE 1:**
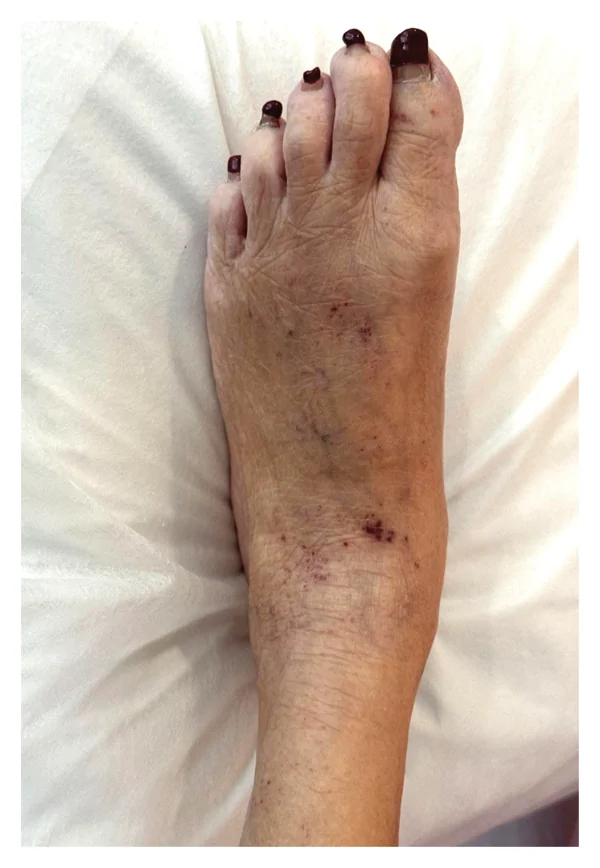
Vesicular eruptions over the left dorsum of the foot and big toe consistent with herpes zoster infection.

Routine hematological and biochemical investigations were within normal limits. Erythrocyte sedimentation rate was mildly raised at 38 mm/hr (reference < 20 mm/hr) and C‐reactive protein at 8.2 mg/L (< 3.0 mg/L).

Magnetic resonance imaging (MRI) of the brain was unremarkable. A noncontrast MRI of the lumbosacral spine demonstrated degenerative spondylotic changes most pronounced at L4‐L5 and L5‐S1, with mild right and moderate left exit foramina narrowing. There was no neural compression to explain the severity of the foot drop.

A lumbar puncture was performed with a normal cerebrospinal fluid (CSF) opening pressure of 18 cm H_2_O. CSF analysis revealed lymphocytic pleocytosis (155 cells/mm3, 80% lymphocytes, and 20% neutrophils), elevated protein of 1.67 g/L, and reduced CSF/serum glucose ratio of 0.47 (CSF glucose 2.7 mmol/L; serum glucose 5.7 mmol/L). CSF culture was negative, and oligoclonal bands were not detected. CSF acid‐fast bacillus (AFB) smear was negative. AFB culture was not performed due to insufficient sample volume. A multiplex meningitis/encephalitis polymerase chain reaction (PCR) panel (BioFire FilmArray) of the CSF was negative for all targets except VZV. VZV DNA was subsequently detected from a skin swab of the foot vesicles, confirming the diagnosis of VZV reactivation.

In view of the acute radicular pain, dermatomal vesicular eruption, lower motor neurone pattern weakness affecting multiple left leg muscle groups, CSF lymphocytic pleocytosis, and positive VZV PCR, a diagnosis of VZV‐associated meningoradiculitis was initially made. After discussion with the infectious diseases team, she was treated with intravenous acyclovir for 2 weeks in accordance with institutional protocol for VZV infection involving the nervous system. She was commenced on analgesia for neuropathic pain. She was then transferred to a rehabilitation ward for intensive physiotherapy and gait training.

A nerve conduction study and electromyography (NCS/EMG) was performed 3 months after symptom onset. This demonstrated a severe left common peroneal neuropathy at the fibular head with sciatic nerve involvement, without electrophysiological evidence of L5 radiculopathy. Needle EMG of the left tibialis anterior revealed acute spontaneous responses with polyphasic discrete motor unit action potentials, suggesting residual axon continuity as well as ongoing renervation.

At five‐month outpatient review follow‐up, there was an improvement in gait, though the neuropathic pain persisted, requiring symptomatic pharmacological therapy.

At nine months, her lower limb strength had returned to full power in all muscle groups except ankle dorsiflexion, which remained at MRC Grade 3. She continued physiotherapy and was referred to occupational therapy for a nighttime ankle foot orthosis.

A timeline from diagnosis to discharge and follow‐up has been illustrated in Table 1.

The combination of an acute painful lumbar syndrome with a predominantly dense unilateral foot drop raised several diagnostic possibilities, which were considered and excluded as follows.

A compressive L5‐S1 radiculopathy or lumbar disc herniation was the initial working diagnosis, given the preceding back pain and the degenerative changes seen on imaging. However, the noncontrast MRI of the lumbosacral spine did not show neural compression proportionate to a complete (MRC Grade 0) foot drop.

A compressive common peroneal palsy at the fibular head, for example, from prolonged bed rest or leg positioning during the acute illness, could not be entirely excluded and may have been contributory; however, it does not account for the preceding dermatomal pain, the CSF pleocytosis, or the positive VZV PCR.

Other causes of lumbosacral plexopathy were considered unlikely. There was no biochemical evidence of diabetes mellitus, making an associated lumbosacral radiculoplexoneuropathy improbable. A neoplastic or infiltrative process was not supported, given the absence of constitutional symptoms, normal hematological and CSF cytology results, and clinical improvement after acyclovir and rehabilitation. An acute inflammatory neuropathy such as Guillain–Barre syndrome was also unlikely, as the deficit was strictly focal and asymmetric, and the CSF showed lymphocytic pleocytosis rather than albuminocytological dissociation.

Finally, a bacterial, tuberculous, or fungal meningitis was considered because of the reduced CSF‐to‐serum glucose ratio, but CSF culture was negative. The multiplex meningitis/encephalitis PCR panel was negative for all six bacterial targets and for *Cryptococcus neoformans/gattii*. CSF AFB smear was negative. AFB culture was not performed due to insufficient sample volume. Tuberculous meningitis was in any case considered unlikely given the absence of fever or constitutional symptoms, the absence of tuberculosis risk factors, the acute rather than subacute course, the only mildly reduced glucose ratio (0.47), and the detection of an alternative pathogen in both CSF and skin.

The detection of VZV DNA in both the CSF and a vesicle swab, together with the clinical response to acyclovir, confirmed and supported VZV as the sole identified etiology.

The NCS/EMG at 3‐month follow‐up localized the lesion to the common peroneal nerve at the fibular head with sciatic nerve involvement rather than to a lumbar nerve root, supporting a predominantly peripheral process rather than root compression.

## 3. Discussion

This case illustrates VZV reactivation presenting as meningoradiculitis with an evolving sciatic and common peroneal neuropathy in an immunocompetent elderly woman, initially mimicking a mechanical lumbar polyradiculopathy. Although VZV reactivation usually presents as a painful dermatomal vesicular eruption (herpes zoster), motor complications are relatively uncommon and are easily missed when the rash is delayed or absent (zoster sine herpete).

The initial clinical syndrome was most consistent with VZV‐associated meningoradiculitis, given the severe radicular pain preceding the rash, dermatomal zoster eruption, lower motor neurone pattern weakness affecting different muscle groups in the left leg, inflammatory CSF abnormalities, and detection of VZV DNA in the CSF.

A literature review identified only seven previously reported cases of VZV‐associated sciatic neuropathy with common peroneal neuropathy (Table 2). Although such motor involvement is classically described in immunocompromised patients, it also occurs in immunocompetent individuals [1–5], and immunosenescence is thought to increase this risk in older adults. In a recent cohort of patients with VZV DNA detected in the CSF, the neurological syndromes and outcomes were heterogeneous [6].

**TABLE 2:** Summary table of reported cases of VZV‐associated sciatic neuropathy with common peroneal neuropathy.

Case report	Patient and risk factors	Neurological syndrome, rash‐weakness temporal relation	Neuroimaging and electrophysiological investigations	Treatment and outcome
Abourazzak et al. 2008 [1]	67‐year‐old woman	Left L5 sciatica with foot drop. A vesicular skin rash appeared 1 week before the sciatica began, and the motor deficit developed one week after the onset of the sciatica	MRI: lumbar MRI revealed isolated hypertrophy of the left L5 root without any signs of radicular compression or herniated disc. EMG: motor involvement of the common peroneal nerve	Treated with valaciclovir, pregabalin, and physical therapy. At six months, she had a partial motor recovery with quasinormal ambulation
Boylu et al. 2010 [2]	68‐year‐old man	Right foot drop due to common peroneal and tibial nerve paralysis. Developed motor weakness 10 days after a herpes zoster rash around the knee and popliteal fossa	NCS/EMG: the common peroneal nerve could not be stimulated. Distal tibial latency was prolonged and compound muscle action potential (CMAP) amplitude diminished. EMG showed denervation in muscles innervated by the tibialis anterior and common peroneal nerves distal to the knee	Initially treated with acyclovir. Three months later, there were persistent severe electromyographic denervation and absent motor responses
Sprenger De Rover et al., 2011 [3]	71‐year‐old man	Right leg and foot weakness. Progressive numbness preceded pain and a vesicular rash on the right buttock/thigh, with significant L4‐L5 motor weakness	MRI: lumbar MRI excluded a stenotic lesion. EMG: supported a lower motor neurone lesion affecting the sciatic nerve proximal to the sciatic notch, with reduced interference in tibialis anterior, gastrocnemius, and semimembranosus	Prescribed oral acyclovir for one week, physical therapy, and a splint. Achieved complete recovery at three months
Gopal et al. 2010 (Case 1) [4]	49‐year‐old man	Left lower limb weakness and foot drop. Motor neuropathy developed two to three weeks after the onset of a painful vesicular skin eruption	MRI: spine MRI revealed early degenerative changes without nerve root compression. NCS: showed involvement of the left common peroneal and posterior tibial nerves with decreased conduction velocity and amplitude.	Treated with physiotherapy (no specific antivirals initially given). Achieved complete recovery after 6–12 weeks
Rota et al., 2016 [5]	76‐year‐old woman	Right foot drop. A tender, vesicular skin eruption appeared prior to the onset of the leg weakness	MRI: MRI neurography revealed enlargement, edema, and abnormal contrast enhancement of the right sciatic nerve. EMG: at five days, showed a reduced recruitment pattern; at four weeks, revealed axonal damage and active denervation in the right sciatic nerve territory	Treated with intravenous acyclovir for 10 days. The clinical picture remained steady, with persistent denervation noted at four weeks
Leo et al. 2009 [11]	79‐year‐old woman on prednisone for temporal arteritis, with a history of prior herpes zoster cases	Sciatica and right foot drop. Sciatic pain was followed four days later by a foot drop, and a vesicular rash appeared two days after the motor weakness began	MRI: not explicitly specified. NCS/EMG: revealed severe right sciatic neuropathy and completely absent motor nerve responses from the right peroneal nerve	Treated with intravenous acyclovir and oral pregabalin. Sciatic pain resolved quickly, but the foot drop showed no improvement at nine months
Allorent et al., 2013 [14]	76‐year‐old woman with a history of Wegener’s granulomatosis, renal impairment, and prior infections, actively taking immunosuppressants	Left L5 sciatica and hemi‐cauda equina syndrome. Back pain onset was followed three weeks later by sciatica, then one week later by a dermatomal rash, and finally motor loss one week after the rash	MRI: showed only degenerative L5‐S1 disk disease. EMG: electrophysiological testing was consistent with radiculitis	Treated with intravenous acyclovir and immunoglobulins and stopped immunosuppressants. At five months, pain and sphincter issues resolved, but motor deficit was unchanged

The neurological complications of VZV are broad, ranging from postherpetic neuralgia to meningoencephalitis and myelitis. Motor involvement, termed zoster paresis or segmental zoster paresis, occurs in a minority of patients [7–9]. In our patient, an acute lower back pain radiating to the leg preceded the rash and led to an initial diagnosis of musculoskeletal strain, consistent with reports that zoster radiculopathy can present with dermatomal pain before the rash and be confused with compressive lumbar disease [10].

The rash may also be atypical. Leo et al. described a 79‐year‐old woman with sciatica and foot drop followed several days later by an atypical petechial rash in the L4‐L5 distribution, underscoring that both the timing and the morphology of the rash may be nonclassical in VZV‐related sciatic neuropathy [11].

A limitation of our case is the use of noncontrast lumbosacral MRI. Even though the appearance of mild degenerative changes and the mismatch between these findings and the severe neurological deficit had prompted further investigations, use of contrast‐enhanced MRI could have potentially revealed enhancement of affected nerve roots, plexus, or peripheral nerves. This would enable a better characterization of the full extent of the neuroanatomy being affected. This is supported by a previous report showing that contrast‐enhanced MRI can demonstrate nerve root or sciatic nerve enhancement in segmental zoster paresis [5].

Neurophysiological testing was deferred during the acute admission for practical and infection control reasons. This is relevant because NCS/EMG performed within the first one to two weeks may be normal or nonlocalizing before denervation is established. The NCS/EMG for our patient at three months nonetheless confirmed a severe left common peroneal neuropathy at the fibular head with sciatic nerve involvement, without electrophysiological evidence of L5 radiculopathy, supporting a predominantly peripheral distribution of established injury rather than root compression.

Herpes zoster radiculopathy that mimics disc herniation both clinically and electrophysiologically has also been described in a previous case report [12]. Similar patterns have been described in case reports of segmental zoster paresis and VZV‐associated lumbosacral radiculoplexoneuropathy [8, 9], where initial radicular pain and imaging findings are followed by electrodiagnostic evidence of plexus or peripheral nerve involvement. We postulated that the initial severe radicular pain in our patient signified the meningeal and nerve root inflammation in the early phase of illness. The subsequent NCS/EMG had only revealed the residual, predominantly peripheral component of the injury and not the initial polyradiculitis after the completion of the course of acyclovir. This final localization with predominantly peripheral nerve involvement also reflects the distribution of established axonal injury, rather than excluding possible earlier proximal or root‐level inflammation.

The profound foot drop reflects the pathophysiology of VZV reactivation in the dorsal root ganglion, with spread to the ventral roots, anterior horn cells, or plexus, producing inflammatory neuritis, demyelination, or neuronal loss. VZV neurotropism can also cause vascular invasion, leading to ischemic neuropathy or perineuritis [13]. Severe lumbosacral VZV involvement can present as sciatica with motor loss and even hemi‐cauda equina syndrome [14]. Corticosteroids have been combined with antivirals to limit this inflammatory response in some reports, although our patient recovered with intravenous acyclovir alone.

The 14‐day course of intravenous acyclovir was selected according to our institutional protocol for VZV infection involving the nervous system. Across reported cases, early antiviral therapy with supportive neurorehabilitation is consistently emphasized, although outcomes vary and residual deficits are common. The early intravenous acyclovir and rehabilitation produced early gait improvement despite residual weakness in our patient. She had persistent ankle dorsiflexion weakness (MRC Grade 3) at nine months follow up, requiring continued physiotherapy and ankle foot orthosis. This underscores that even with timely treatment, VZV infection can result in long term focal deficits, as reported in cases of segmental zoster paresis [5].

In summary, VZV should be considered in patients with acute radicular pain and focal motor deficits that are disproportionate to imaging, even when a rash is absent or delayed. Early antiviral therapy may limit irreversible damage and aid recovery, although residual weakness and neuropathic pain can persist.

## Author Contributions

Yi Ru Tan and Samuel Zheng Fong Goh conceptualized the study, collected the data, and drafted the article. Yi Ru Tan is responsible for the images. Yi Ru Tan, Samuel Zheng Fong Goh, Yeow Hoay Koh, and Gabriel Shih Chung Yiin revised the manuscript and approved the final copy.

## Funding

No funding was received for this work.

## Ethics Statement

Written informed consent for publication and permission for the use of clinical photographs was obtained from the patient.

The case has been sufficiently anonymized in accordance with ICMJE recommendations, and no identifying patient information is included.

Institutional approval for publication was obtained from Changi General Hospital, in accordance with local publication governance requirements.

## Consent

Please see the Ethics statement.

## Conflicts of Interest

The authors declare no conflicts of interest.

## Data Availability

No datasets were generated during the current study.
